# Factors associated with the mental health of back-to-Wuhan university students based on quantile regression model during the COVID-19 period

**DOI:** 10.1186/s12888-022-03828-z

**Published:** 2022-04-21

**Authors:** Qian Wu, Lijun Zhuo, Hao Li, Ling Zheng, Guoqing Ma, Hongbing Tao

**Affiliations:** School of Medicine and Health Management, Tongji Medical College, Huazhong University of Science and Technology, Wuhan, 430030 China

**Keywords:** COVID-19, Mental health, University students, Quantile regression analysis, Discrimination

## Abstract

**Background:**

The COVID-19 pandemic had brought the increased levels of depression and anxiety on people. Our study investigated the levels of mental health and influencing factors among back-to-Wuhan university students.

**Methods:**

A cross-sectional questionnaire survey was conducted from 31 August 2020, to 14 September 2020 by convenience sampling on the back-to-Wuhan university students, which included the Generalized Anxiety Disorder Scale (GAD-7), Patient Health Questionnaire-9 (PHQ-9), the Insomnia Severity Index-7 (ISI-7), the revised Impact of Event Scale (IES-R) scales, and the basic demographic characteristics. Moreover, quantile regression analysis was used to identify the key factors related to the mental health variables of the back-to-Wuhan university students during the COVID-19 period.

**Results:**

The results from 1017 participants suggested that the prevalence rates of the anxiety, depression, insomnia, and distress were 44%, 47.5%, 37.7%, 57.7%, respectively. Quantile regression showed that mental health scores were negatively associated to age, years from graduation, being discriminated against owing to the experience in Wuhan, and the attitude on the future of COVID-19, while was positively related to the education level (*P* < 0.05). Especially, the education level was highly related with anxiety (25th = 1.64, 50th = 2.54).

**Conclusion:**

The finding showed that the respondents who were near graduation, discriminated owing to the experience in Wuhan, and worried about the future trend of COVID-19 had a higher risk of negative psychologic status, especially in the bottom and median quantile, and might require more psycho-social interventions or support.

## Background

The novel Coronavirus 2019(COVID-19) which was first reported in Wuhan, Hubei province, China in late December 2019 was transmitted by respiratory tract and aerosol, and have had a huge impact on the world [1]. Owing to the successful response, including the quarantine measures lasted for more than two months and effective contact tracing, the number of new confirmed cases and suspected cases in China have gradually reduced, and the pandemic in China was under control [2, 3]. Unfortunately, it wasn’t the case in the other countries. As of Nov27, 2021, the World Health Organization announced that the number of cumulative confirmed cases worldwide is nearly 259 million, and the cumulative dead patients are 5.18 million. [4] Previous studies have showed that large-scale, unexpected public health events such as the severe acute respiratory syndrome (SARS) epidemic in China in 2003 [5, 6] and the Middle East respiratory syndrome coronavirus in 2014 [7, 8], not only threatened people’s physical health, but also caused serious harm to people’s psychology [9], and this psychological impact may last for a long time [10, 11].

Previous cross-sectional studies implicated that the COVID-19 crisis triggered mental health problems of general people, including the medical staff, patients, the elderly and children, even in post-crisis period [12]. Psychological distress associating with demographic factors among medical staffs were established by investigating 1,563 and 12,759 samples respectively, and the results showed that the detection rates of depression, anxiety, insomnia and stress symptoms were higher than the public psychological abnormality examination rate during the COVID-19 epidemic. Psychological distress associating with demographic factors among medical staffs were established by investigating 1,563 and 12,759 samples respectively, and the results showed that the detection rates of depression, anxiety, insomnia and stress symptoms were higher than the public psychological abnormality examination rate during the COVID-19 epidemic [13–15]. Similarly, the COVID-19 had serious repercussions for the mental health of the discharged patients, whose proportion of moderate to severe anxiety was 10.4%, and severe depression was 19% [16]. The elderly and children, as vulnerable groups, also suffered from serious mental health problems [17].

The result that SARS-related mental health problems associating with demographic factors among college students during the SARS epidemic has been proven [18]. The same to SARS period, university students in Guangzhou, China had mental distress caused by fear of hemagglutinin type-1 neuraminidase type-1 influenza virus (H1N1) during the H1N1period [19]. The severity of COVID-19 far exceeds the severity of SARS and the H1N1 crisis, while, the mental health of university students in post-COVID-19 era have not yet been explored, especially the university students in Wuhan. The students in Wuhan, the epicenter of the COVID-19 pandemic, regarded as carriers of the virus were discriminated against, causing the stress, anxiety, and fear problems [20]. Additionally, a large number of universities have postponed the beginning date of new term and adopted the E-learning model [21]. While preliminary evidence illustrated that the home-quarantined college students worried about the COVID-19 were supposed to affect their mental health. 24.9% of 7,143 college students in home isolation suffered from mild anxiety and Above [22]. Previous research suggested that there would be a long-term negative impact on people’s psychological health in a public health crisis. Hence, the impact factors should be explored actively [23]. And some preventive measures, including mental health education and promotion were warranted to alleviate the mental distress.

Hundreds of COVID-19 confirmed cases reported, while American students returned to college campuses without keeping suitable social distance from others [24].In September 2020, a large number of university students returned to school in Wuhan. To reduce the risk of infection, closed management measures, including students "unnecessarily not going out", wearing masks and maintaining social distance were put into effect, which might influence the study life and psychological well-being of back-to-Wuhan university students. Previous research showed that there were more suicidal behaviors among college students with post-traumatic stress and poor mental health, especially for female college students [25]. Previous researches suggested that measuring and evaluating the mental health of college students are conducive to monitor mental health diseases and behavioral problems, and being able to predict and intervene in behavior changes [26, 27]. Therefore, it is necessary to pay attention to the mental health of back-to-Wuhan university students, identify key impact factors and implement efficient measures as soon as possible [28].

For universities, how to guide the students to deal with the public health emergencies, control their feelings and avoid sinking into negative emotions have become an issue of far greater momentousness and urgency to solve. Thus, during the COVID-19 epidemic, this current research surveyed mental health and influence factors among back-to-Wuhan university students makes sense.

The aims of this study are:(i) to analyze the mental health of back-to-Wuhan university students in Wuhan during the post-COVID-19 epidemic period; (ii) to find the key impact factors of mental health variables and then to provide a certain basis of preventing measures to the mental health of university students.

## Methods

### Study design

This research was a crossed-sectional survey for university students. All mental health variables, including anxiety, depression, insomnia, and distress were measured by the online questionnaire tool ‘Questionnaire Star Application’. Ethical approval was obtained from the Ethics Committee of Tongji Medical College, Huazhong University of Science and Technology (No: IORG0003571).

Convenience sampling was conducted to collect data from 31 August 2020, to 14 September 2020 in Wuhan. The purpose and content of this survey had been explained to each respondent, and the digital informed consent of this research had been provided to all respondents. Each questionnaire was filled out anonymously.

### Participants

The questionnaires of participants were completed online from different universities on account of the closed management, the policy of university students "unnecessarily not going out". The inclusion criteria were: (i) university students covering undergraduate, master and doctoral candidate, who studied in Wuhan before the COVID-19 outbreak and currently; (ii) 18 years old and above.

The formula of the target participant sample size is following *N* = Z_α_^2^P(1-P)/ δ^2^, (α = 0.05, Z_α_ = 1.96, P means the positive rate of mental health problem), and the absolute allowable error of ratio estimate δ was 0.05. The positive rate is 41.1% reported at anxiety symptoms and 26.3% demonstrated at depressive in the previous study [29, 30]. For the reduce of sampling error, we expanded the sample size by 100% with the goal of participants at least 744 completed questionnaires. A total of 1,332 back-to-Wuhan university students were surveyed, 192 people refused to participate, and 1,017 valid samples were included, with an effectivity rate of 76.35%.

### Measures

The general questionnaire mainly included two parts. The first part was sociodemographic information, such as age, gender, department category of the university, hometown, years from graduation, whether you have been discriminated against because of studying in Wuhan, and whether COVID-19 rebound again in China.

The second part was mental health variables, including anxiety, depression, insomnia, and distress, measured by the Generalized Anxiety Disorder Scale (GAD7) ranging 0–21, the Patient Health Questionnaire (PHQ-9) ranging 0–27, the Insomnia Severity Index (ISI-7) ranging 0–28, and the Impact of Event Scale–Revised (IES-R) respectively. These scales had good reliability and validity in previous studies [29, 31–33]. The scores of these scales were classified as follows: GAD-7, normal (0–4), mild (5–9), moderate (10–13), moderate to severe (14–18), and severe (19–21); PHQ-9,normal (0–4), mild (5–9), moderate to severe (10–14), and severe (20–27); ISI-7, normal (0–7), mild (8–14), moderate (15–21), severe (22–28); IES-R, normal (0–7), mild (8–14), moderate (15–21) and severe (22–28). The Cronbach’s alpha coefficients of the Chinese versions of PHQ-9, GAD-7, ISI-7 and IES-7 were 0.929, 0.918, 0.903 and 0.899, respectively in this study. Therefore, the internal consistency was excellent in the current study.

### Statistical analysis

Stata statistical software version 15.0 (StataCorp LLC, Texas, USA) was used to analyze the data. First, sociodemographic characteristics were illustrated by quantity and frequency. Normality distributions test was used for dependent variables. Pearson’s correlations were performed to test the correlation between the influence factors and mental health variables. Secondly, the Kruskal–Wallis test was applied for the difference comparison among groups, in which the prevalence rate of anxiety (GAD7 ≥ 5 points), depression (PHQ 9 ≥ 5 points), insomnia (ISI 7 ≥ 8 points), distress (IES 7 ≥ 8 points) could be evaluated, respectively. Finally, quantile regression was used to explore regression relationship of mental health variables and sociodemographic characteristics. As appropriate, with *P* less than 0.05 considered to be statistically significant. Moreover, considering the distribution characteristics of the dependent variables, the P_25_, P_50_, P_75_ conditional quantile points were selected as representatives to describe the quantile regression analysis results. The better group (represented by P_25_ quantile points), general group (represented by P_50_ quantile points), and poor group (represented by P_75_ quantile points) of each dependent variable showed the sociodemographic characteristics related to anxiety, depression, insomnia, and distress.

## Results

### Sociodemographic characteristics

As is revealed in Table 1, a total of 1017 back-to-Wuhan university students participated in the questionnaire survey. Among them, 475 (46.7%) are males, and more than 92.8% participants are between 18–25 years old. The number and proportion of undergraduates, postgraduates, doctors are 490 (48.2%), 447 (44%),80 (7.9%), respectively. 363 (35.7%) are medical students. There are 684 (67.2%) students with less than two years left before graduation. Only 41 (4%) of the university students surveyed are from Wuhan, 283 (27.8%) students are from Hubei Province except for Wuhan, and 693 (68.1%) students from other provinces. Most students [604 (59.4%)] have been discriminated against because of their experience in Wuhan. 258 (25.4%) respondents are optimistic about the COVID-19 epidemic, 322 (31.7%) respondents believe that the epidemic might rebound.

**Table 1:** The sociodemographic characteristics of participants

	Total	%
Total	1017	100
Sex		
Male	475	46.7
Female	542	53.3
Age		
18–21	431	42.4
22–25	513	50.4
26–29	63	6.2
≥ 30	10	1
Highest Education level		
Undergraduate student	490	48.2
Master student	447	44
PHD student or above	80	7.9
Department category of the university		
Department of Medicine-related	363	35.7
Department of Non-Medicine-related	654	64.3
Years from graduation		
Within 1 year	348	34.2
Within 2 year	336	33
Within 3 year	242	23.8
4 years and above	91	8.9
Hometown		
Wuhan	41	4
Hubei except for Wuhan	283	27.8
Other provinces	693	68
Whether Be discriminated		
Yes	413	40.6
No	604	59.4
COVID-19 be rebounding again		
Yes	322	31.7
No	258	25.4
Uncertain	437	43

### The distribution and correlation among variables

Table 2 presents the score distributions of GAD-7, PHQ-9, ISI-7, IES-R scales. From the skewness, kurtosis and extreme value distribution ratio, the scores of each dimension do not conform to the normal distribution (*P* < 0.01).

**Table 2:** The distribution characteristics of mental health variables

	Mean	Standard deviation	Median	Mode	Skewness	Kurtosis	Normal-proportion	Severe- proportion	Normality
GAD-7	4.36	4.3	4	0	1.04	0.88	0.56	0.05	< 0.01**
PHQ-7	5.18	5.18	4	0	1.12	1.1	0.53	0.06	< 0.01**
ISI-7	6.76	5.49	6	0	0.87	0.31	0.62	0.11	< 0.01**
IES-R	8.75	5.01	8	8	0.39	0.11	0.42	0.13	< 0.01**

*Note. GAD-7* 7-item General Anxiety Disorder Scale, *PHQ-9* 9-item Patient Health Questionnaire (depression), *ISI-7* 7-item Insomnia Severity Index Scale, *IES-R* the Impact of Event Scale–Revised (distress)

^**^P < 0.01 *P < 0.05

Table 3 provides the correlations between the sociodemographic characteristics and the mental health variables, and reflects the positive and negative correlations significantly.The results show that anxiety symptom is negatively correlated with years from graduation (*r* = -0.07, *P* < 0.05), whether be discriminated (*r* = -0.09, *P* < 0.01) and COVID-19 be rebounding (*r* = -0.10, *P* < 0.01), and all three variables are also negatively correlated with the depression, insomnia and distress. The depression symptom is positively correlated with age (*r* = 0.10, *P* < 0.01). The insomnia is positively correlated with age (*r* = 0.18, *P* < 0.01) and highest education level (*r* = 0.16, *P* < 0.01), while the distress symptom is negatively correlated with the department category of the university.

**Table 3:** Pearson’s correlations among variables

Variable	1	2	3	4	5	6	7	8
1.Gender	-							
2.Age	0.10^**^	-						
3.Highest Education level	0.08^*^	0.76^**^	-					
4.Department category of the university	-0.19^**^	-0.17^**^	-0.10^**^	-				
5.Years from graduation	-0.04	-0.20^**^	-0.002	-0.09^**^	-			
6.Hometown	-0.03	-0.03	< 0.001	0.03	0.07^*^	-		
7.Whether be discriminated	-0.04	-0.11^**^	-0.10^**^	0.06	0.05	-0.22^**^	-	
8.COVID-19 be rebounding again	0.13^**^	-0.04	-0.03	0.04	-0.03	0.06^*^	0.06	-
9.GAD-7	0.01	0.04	0.04	-0.03	-0.07^*^	0.02	-0.09^**^	-0.10^**^
10.PHQ-9	0.05	0.10^**^	0.05	-0.02	-0.08^**^	-0.01	-0.09^**^	-0.07^*^
11.ISI-7	0.04	0.18^**^	0.16^**^	-0.02	-0.07^*^	-0.01	-0.10^**^	-0.08^*^
12.IES-R	-0.002	0.09^**^	0.05	-0.06^*^	-0.12^**^	-0.004	-0.17^**^	-0.08^**^

*Note*. *GAD-7* 7-item General Anxiety Disorder Scale, *PHQ-9* 9-item Patient Health Questionnaire (depression), *ISI-7* 7-item Insomnia Severity Index Scale, *IES-R* the Impact of Event Scale–Revised (distress)

^**^P < 0.01 *P < 0.05

### The severity of measurements and associated factors

As shown in Table 4, there is quite a large proportion of respondents with symptom of anxiety [447 (44%)], depression [483 (47.5%)], insomnia [383 (37.7%)], and distress [587 (57.7%)]. Kruskal–Wallis test demonstrates that the prevalence rates of anxiety, insomnia and distress varies among different ages, levels of education, whether the university students were discriminated owing to the experience in Wuhan (*P* < 0.05). Also, the opposite attitude to the future trend of the COVID-2019 epidemic has led to the different infection rates of anxiety and distress (*P* < 0.05). For whom were discriminated due to the experience in Wuhan, they are easily to develop the psychological symptoms of depression (*P* < 0.05). Simultaneously, whether the participants suffering from the distress are affected by sex, year from graduation, and the attitude to the future trend of the COVID-2019 (*P* < 0.05).

**Table 4:** The prevalence rates under different sociodemographic characteristics

	GAD-7			PHQ-9			ISI-7			IES-R		
	< 5	≥ 5	*p*-Value	< 5	≥ 5	*p*-Value	< 8	≥ 8	*p*-Value	< 8	≥ 8	*p*- Value
Total	570(56)	447(44)		534(52.5)	483(47.5)		634(62.3)	383(37.7)		430(42.3)	587(57.7)	
Gender												
Male	270(47.4)	205(45.9)	0.633	265(49.6)	210(43.5)	0.050	308(48.6)	167(43.6)	0.123	199(46.3)	276(47)	0.694
Female	300(52.6)	242(54.1)		269(50.4)	273(56.5)		326(51.4)	216(56.4)		231(53.7)	311(53)	
Age												
18–21	262(46)	169(37.8)	0.024*	245(45.9)	186(38.5)	0.018*	304(47.9)	127(33.2)	< 0.001**	205(47.7)	226(38.5)	0.002**
22–25	266(46.7)	247(55.3)		254(47.6)	259(53.6)		297(46.8)	216(56.4)		199(46.3)	314(53.5)	
26–29	37(6.5)	26(5.8)		31(5.8)	32(6.6)		29(4.6)	34(8.9)		23(5.3)	40(6.8)	
≥ 30	5(0.9)	5(1.1)		4(0.7)	6(1.2)		4(0.6)	6(1.6)		3(0.7)	7(1.2)	
Education level												
Undergraduate student	291(51.1)	199(44.5)	0.043*	275(51.5)	215(44.5)	0.064	337(53.2)	153(39.9)	< 0.001**	223(51.9)	267(45.4)	0.026*
Master student	237(41.6)	210(47)		215(40.3)	232(48)		255(40.2)	192(50.1)		175(40.7)	272(46.3)	
PHD student or above	42(7.4)	38(8.5)		44(8.2)	36(7.5)		42(6.6)	38(9.9)		32(7.4)	48(8.2)	
Department category of the university												
Department of Medicine-related	195(34.2)	168(46.3)	0.265	183(34.3)	180(37.3)	0.319	228(36)	135(35.2)	0.818	139(32.3)	224(38.2)	0.072
Department of Non-Medicine-related	375(65.8)	279(62.4)		351(65.7)	303(62.7)		406(64)	248(37.9)		291(67.7)	363(61.8)	
Years from graduation												
Within 1 year	186(32.6)	162(36.2)	0.123	171(32)	177(36.6)	0.053	205(32.3)	143(37.3)	0.092	124(28.8)	224(38.2)	0.002**
Within 2 year	185(32.5)	151(33.8)		174(32.6)	162(33.5)		211(33.3)	125(32.6)		143(33.3)	193(32.9)	
Within 3 year	147(25.8)	95(21.3)		138(25.8)	104(21.5)		160(25.2)	82(21.4)		120(27.9)	122(20.8)	
4 years and above	52(9.1)	39(8.7)		51(9.6)	40(8.3)		58(9.1)	33(8.6)		43(10)	48(8.2)	
Hometown												
Wuhan	19(3.3)	22(4.9)	0.361	18(3.4)	23(4.8)	0.323	28(4.4)	13(3.4)	0.982	18(4.2)	23(3.9)	0.674
Hubei except Wuhan	171(30)	112(25.1)		161(30.1)	122(25.3)		173(27.3)	110(28.7)		116(27)	167(28.4)	
Other provinces	380(66.7)	313(70)		355(66.5)	338(79)		433(68.3)	260(67.9)		296(68.8)	397(67.6)	
Whether Be discriminated												
Yes	216(37.9)	197(44.1)	0.047*	198(37.1)	215(44.5)	0.016*	233(36.8)	180(47)	0.001**	134(31.2)	279(47.5)	< 0.001**
No	354(62.1)	250(55.9)		336(62.9)	268(55.5)		401(63.2)	203(53)		296(68.8)	308(52.5)	
The COVID-19 whether rebound again in China												
Yes	157(27.5)	165(36.9)		154(28.8)	168(34.8)		190(30)	132(34.5)		110(25.6)	212(36.1)	
No	160(28.1)	98(21.9)	0.027*	149(27.9)	109(22.6)	0.261	164(25.9)	94(24.5)	0.175	128(29.8)	130(22.1)	0.038*
Uncertain	253(44.4)	184(41.2)		231(43.3)	206(42.7)		280(44.2)	157(41)		192(44.7)	245(41.7)	

*Note*. *GAD-7* 7-item General Anxiety Disorder Scale, *PHQ-9* 9-item Patient Health Questionnaire (depression), *ISI-7* 7-item Insomnia Severity Index Scale, *IES-R* the Impact of Event Scale–Revised (distress)

^**^P < 0.01 *P < 0.05

Table 5 reports the related effects for the25th, 50th, and 75 quantiles of the distribution of the anxiety, depression, insomnia and distress. Further, age, years from graduation, being discriminated against owing to the experience in Wuhan, and the attitude on the future of COVID-19 are negatively associated to the mental health scores, while the education level is positively related to it. First, the column (1) reveals the significant positive effect of education level wildly across anxiety, 0.364 in the 25th quantile changing to 1.270 in the 50th quantile, but the negative effect of the uncertainty on the future trend of COVID-19(25th = -0.546,50th = -0.750), indicating that university students with less anxiety are more susceptible. Second, in column (2), the heavier the scientific research tasks, the more depression on the participants, changing from 0.750 in the 25th quantile to 1.000 in the 75th quantile. Third, columns 3–4 of Table 5 presents that those are significant and negative at 25th, 50th, and 75th quantiles and the university students in graduation grade developed insomnia (25th = -0.528,75th = -0.667) and distress (25th = -0.333,75th = -1.417).

**Table 5:** Different quantiles analysis of mental health and associated factors

	**(1) GAD-7**			**(2) PHQ-9**			**(3) ISI-7**			**(4) IES-R**		
	P_25_	P_50_	P_75_	**P**_**25**_	**P**_**50**_	**P**_**75**_	**P**_**25**_	**P**_**50**_	**P**_**75**_	**P**_**25**_	**P**_**50**_	**P**_**75**_
**Gender**	0.091 (0.35)	0.248 (0.53)	-0.327 (-0.73)	-0.765 (-0.57)	0.250 (0.55)	0.571 (0.87)	0.250 (0.59)	-0.333 (-0.95)	-0.667 (-1.68)	0.154 (0.59)	-0.333 (0.63)	0.583 (1.09)
**Age**	-1.000 (-1.85)	-2.250 (-2.34)	-0.523 (-0.71)	-0.083 (-0.09)	1.000 (1.06)	2.000 (1.48)	0.083 (0.09)	0.667 (0.92)	0.333 (0.41)	0.111 (0.14)	-0.333 (-0.31)	-1.08 (-0.99)
**Highest Education level**	0.364* (1.64)	1.270* (2.54)	0.250 (0.86)	0.750* (2.04)	1.000** (2.59)	1.571** (2.84)	0.333 (0.93)	-0.277 (-0.47)	-0.416 (-0.72)	0.444 (1.39)	0.333 (0.75)	0.500 (1.11)
**Department category of the university**	-0.273 (-1.00)	-0.500 (-1.03)	-0.275 (-0.70)	-0.250 (-0.55)	-0.250 (-0.53)	0.714 (1.05)	-0.667 (-1.51)	-1.000** (-2.73)	-0.667 (-1.62)	-0.444 (-1.13)	-0.333 (0.61)	-0.083 (-0.15)
**Years from graduation**	-0.091 (-0.68)	-0.500* (-2.12)	-0.235 (-1.44)	-0.146 (-1.13)	-0.250 (-1.08)	-0.143 (-0.43)	-0.528* (-2.32)	-0.667** (-3.74)	-0.667** (-3.33)	-0.333* (-1.74)	-0.667* (-2.50)	-0.583* (-2.16)
**Hometown**	-0.455 (-0.70)	-2.360 (-1.73)	-0.259 (-0.29)	0.167 (0.15)	0.250 (0.22)	-0.286 (-0.18)	0.500 (0.47)	-0.386 (-0.69)	0.603 (0.75)	-0.556 (-0.59)	-1.667 (-1.28)	-1.417 (-1.07)
**Be discriminated or not**	-0.546* (-2.09)	-0.500 (-1.08)	-0.250 (-0.73)	-0.667 (-1.54)	-0.750 (-1.65)	-1.571* (-2.41)	-1.750** (-4.14)	-1.667** (-4.76)	-1.667** (-4.23)	-0.556 (-1.48)	-1.667** (-3.18)	-0.917 (-1.73)
**COVID-19 be rebounding again or not**	-0.546** (-3.64)	-0.750** (-2.81)	-0.370 (-1.27)	-0.417 (-1.67)	-0.148 (-0.96)	-0.429 (-1.14)	-0.417 (-1.71)	-0.333 (-1.66)	-0.333 (-1.47)	-0.444* (-2.05)	-0.333 (-1.10)	-0.333 (-1.09)
**Constant**	4.909** (2.82)	12.000** (3.87)	9.750** (4.27)	5.000 (1.72)	5.000 (1.65)	7.286 (1.67)	9.333** (3.30)	14.667** (6.27)	16.000** (6.08)	4.778 (1.90)	12.000** (3.42)	13.917** (3.93)

*Note*. *GAD-7* 7-item General Anxiety Disorder Scale, *PHQ-9* 9-item Patient Health Questionnaire (depression), *ISI-7* 7-item Insomnia Severity Index Scale, *IES-R* the Impact of Event Scale–Revised (distress)

^**^P < 0.01 *P < 0.05

## Discussion

The results of the survey of 1,017 back-to-Wuhan university students indicated that the prevalence rates of anxiety, depression, insomnia, and distress were 44%, 47.5%, 37.7%, and 57.7%, respectively, which were higher than those during the non-epidemic periods [34, 35]. Previous studies have shown that the psychological health of university students under the influence of public health emergencies to a large extent [17, 36, 37], on account of the university students’ easier access to information related to the pandemic [38, 39], lack of experience in responding to public health emergencies, and their studies and future career development affected by the COVID-19 [34].It has been demonstrated that the increasing number of confirmed cases, suspected cases, the outbreak rebounded in other provinces or countries [40] and the continuous negative news reports about the COVID-19 [41], have induced people’s anxiety, depression.

However, the gender on the prevalence rate was not significant difference, keeping with the previous research finding [22, 42]. But the majority of surveys conducted previously found the significant difference among the different gender, such as American male college students are easily depressed than female [43], on the contrary to Alansari’s research in Saudi Arabia [44],which might be caused by the narrowing of the gap of people’s access to education and information currently. The traditional views of many emotional women under the peer pressure and high expectation from society and family, might increase the risk of depression as seen in other COVID-19 study as well [39].

The result manifested the prevalence rates of anxiety, insomnia, and distress were significant with different ages and educational level of university students, deriving from the greater pressure of scientific research of the master’s and doctoral students, longer home isolation time, worse scientific research environment, and lower efficiency. There were no system online psychological, academic, and employment guidance courses for university students. Similar studies have indicated that some household income might be affected, consequently, some university students could concern sources for tuition due to the outbreak of the pandemic [45].

In accordance with the hypothesis, the quantile regression confirmed the degree of education level has a positive influence on depression. It reports that those suffering more from anxiety could worry about the negative influence of COVID-19 on their academics and science research, indicating that the degree of education level is a sensitive indicator that affects the score of depression. The impact of years from graduation on insomnia and distress is negative, and the impact gradually becomes larger in the median and upper quantiles. The higher education level and the closer to graduation, the more anxiety, depression, insomnia, and distress the participants have. It might result from that limited ways of pressure release under closed management have affected the academic or employment development of university students near graduation.

Furthermore, it also classified and analyzed whether university students had ever been discriminated against due to the experience in Wuhan after returning to school, and the attitude on the future trend of COVID-19. Due to experience related Wuhan city, some discrimination had also affected the mental health of university students to a certain extent, which is consistent with previous research results [46]. The results showed that there were differences in the level of mental health among university students with discrimination and worried about the rebound of the epidemic. It might be related to the stigmatization of Wuhan in the early stage of the epidemic [47]. At the same time, news of repeated fluctuations in the epidemic situation in Dalian, Beijing and other places would also affect the feeling of university students.

Although telephone and online chat psychiatric treatment were advocated during the COVID-19 pandemic [48], the universities should also pay more attention to the mental health of back-to-Wuhan university students, especially in Wuhan. At present, the psychological counseling centers for university students are passive, and they should actively carry out psychological preventions for students near graduation to understand the reasons behind and help them get out of their difficulties. This research innovatively chooses to investigate the mental health of university students returning to school in Wuhan in the post-epidemic period and proposes key associated factors of mental health, which is conducive to maintain the mental health of university students and provide education and management.

The current study has several limitations. First, quantile regression could capture the heterogeneous associations between mental health and explanatory variables, however, the cross-sectional survey was hard to deduce causal inference and had a certain amount of information offset. A longitudinal study could be performed in the further study. Secondly, the research only studied the mental health of back-to-Wuhan university students, and did not cover other parts of China, such as Beijing, Guangzhou, etc., which rebounded in a small range in the post-epidemic period. Third, owing to the unavailable sampling framework and close-off management of back-to-Wuhan university students, convenient sampling was used to collect data, which could not represent the interest population and existed bias. The sample size is not sufficiently representative, which may have a certain impact on the results. Then, the mental health variables were measured by maturity scales, while, self-report instruments might respond bias.

## Conclusion

In conclusion, the findings of this crossed-sectional study suggested that COVID-19 caused the high prevalence rate of the back-to-Wuhan university students on depression, anxiety, insomnia, and distress syndrome, relatively. The back-to-Wuhan university students near graduation, being discriminated against owing to the experience in Wuhan city and worrying about the future trend of COVID-19 were correlated with a higher risk of developing depression and anxiety symptoms than others. These effects were stronger in the bottom and median quantile of anxiety and distress. Moreover, the long-term psychological effects covering causal inference and mediation analysis will help specify the impact of mental health in this population are worth further research.

## Data Availability

The data presented in this study are available on request from the corresponding author. The data are not publicly available due to privacy.

## Abbreviations

COVID-19: Coronavirus disease-2019
GAD-7: Generalized Anxiety Disorder Scale
PHQ-9: Patient Health Questionnaire-9
ISI-7: Insomnia Severity Index-7
IES-R: Revised Impact of Event Scale
SARS: Severe acute respiratory syndrome coronavirus
H1N1: Hemagglutinin type-1 Neuraminidase type-1 influenza virus

## References

[1] de Lima CVC, Candido EL, da Silva JA, Albuquerque LV, Soares LD, do Nascimento MM, de Oliveira SA, Neto MLR. Effects of quarantine on mental health of populations affected by Covid-19. J Affect Disord. 2020;275:253–254. 10.1016/j.jad.2020.06.063 10.1016/j.jad.2020.06.063 PMC7361081 32734916

[2] Li X, Lu P, Hu L, Huang T, Lu L (2020). Factors associated with mental health results among workers with income losses exposed to COVID-19 in China. Int J Environ Res Public Health.

[3] Lin X, Rocha ICN, Shen X, Ahmadi A, Lucero-Prisno D (2021). Challenges and strategies in controlling COVID-19 in Mainland China: lessons for future public health emergencies. J Soc Health.

[4] Coronavirus disease (COVID-19) pandemic https://www.who.int/emergencies/diseases/novel-coronavirus-2019

[5] Lu YC, Shu BC, Chang YY, Lung FW (2006). The mental health of hospital workers dealing with severe acute respiratory syndrome. Psychother Psychosom.

[6] Zhang Q, Huang XQ, He XB, Yang XL, Yu SY (2004). Comparing high-risking SARS medical members' attitude and their mental health characters during the epidemic outbreak of SARS in Beijing. Int J Psychol.

[7] Park JS, Lee EH, Park NR, Choi YH (2018). Mental health of nurses working at a government-designated hospital during a MERS-CoV Outbreak: a cross-sectional study. Arch Psychiatr Nurs.

[8] Park HY, Park WB, Lee SH, Kim JL, Lee JJ, Lee H, Shin HS (2020). Posttraumatic stress disorder and depression of survivors 12 months after the outbreak of Middle East respiratory syndrome in South Korea. BMC Public Health.

[9] Hall RCW, Hall RCW, Chapman MJ (2008). The 1995 Kikwit Ebola outbreak: lessons hospitals and physicians can apply to future viral epidemics. General Hospital Psychiatry.

[10] Maunder RG (2009). Was SARS a mental health catastrophe?. General Hospital Psychiatry.

[11] Usher K, Bhullar N, Jackson D (2020). Life in the pandemic: social isolation and mental health. J Clin Nurs.

[12] Campion J, Javed A, Sartorius N, Marmot M (2020). Addressing the public mental health challenge of COVID-19. Lancet Psychiatry.

[13] Liu S, Yang L, Zhang C, Xiang Y-T, Liu Z, Hu S, Zhang B (2020). Online mental health services in China during the COVID-19 outbreak. Lancet Psychiatry.

[14] Chen Gui-mei RJ, He Xinran, Yuan Hongxu, Chen Ren, Wang Li, Xu Wangquan, Zheng Tao, Ding Hong. Mental health status of medical staff based on SCL-90 in Anhui Province during the epidemic situation of COVID-19. Chinese Journal Disease Control&Prevention. 2020;24(8):(in Chinese). 10.16462/j.cnki.zhjbkz.2020.08.020.

[15] Song Feifei WX, Ju Zhiying, Liu Aixiang, Liu Junjie, Wang Tao. Mental health status and related influencing factors during the epidemic of coronavirus disease 2019 (COVID-19). Hubei J of Preve Med. 2020;31(2):(in Chinese). 10.3969/j.issn.1006-2483.2020.02.006.

[16] Liu D, Baumeister RF, Veilleux JC, Chen C, Liu W, Yue Y, Zhang S (2020). Risk factors associated with mental illness in hospital discharged patients infected with COVID-19 in Wuhan. China. Psychiatry Res.

[17] Yang Y, Li W, Zhang QG, Zhang L, Cheung T, Xiang YT (2020). Mental health services for older adults in China during the COVID-19 outbreak. Lancet Psychiatry.

[18] Main A, Zhou Q, Ma Y, Luecken LJ, Liu X (2011). Relations of SARS-related stressors and coping to Chinese college students' psychological adjustment during the 2003 Beijing SARS epidemic. J Couns Psychol.

[19] Gu J, Zhong Y, Hao Y, Zhou D, Tsui H, Hao C, Gao Q, Ling W, Lau JT (2015). Preventive behaviors and mental distress in response to H1N1 among university students in Guangzhou China. Asia Pac J Public Health.

[20] Zhai Y, Du X (2020). Mental health care for international Chinese students affected by the COVID-19 outbreak. Lancet Psychiatry.

[21] Gewin V (2020). Into the digital classroom Five tips for moving teaching online as COVID-19 takes hold. Nature.

[22] Cao W, Fang Z, Hou G, Han M, Xu X, Dong J, Zheng J (2020). The psychological impact of the COVID-19 epidemic on college students in China. Psychiatry Res.

[23] Ren FF, Guo RJ (2020). Public mental health in post-COVID-19 era. Psychiatria Danubina.

[24] Hundreds of COVID-19 cases reported as students return to college campuses https://edition.cnn.com/2020/08/18/health/colleges-return coronavirus-cases-wellness/index.html

[25] Hwang M (2019). The effects of post-traumatic stress of college students on suicidal behavior: the mediating effect and gender differences of mental health. Int J humanit Soc Sci.

[26] Lee J, Jeong HJ, Kim S (2021). Stress, anxiety, and depression among undergraduate students during the COVID-19 pandemic and their use of mental health services. Innovative Higher Education.

[27] Liu CH, Pinder-Amaker S, Hahm HC, Chen JA. Priorities for addressing the impact of the COVID-19 pandemic on college student mental health. J Am Coll Health. 2020. 10.1080/07448481.2020.1803882. 10.1080/07448481.2020.1803882 PMC8041897 33048654

[28] Lu Q. Basic Intervention on Stress induced by SARS Epidemic. Chinese Mental Health Journal. 2003;17(8):534-535(in Chinese).

[29] Fu W, Yan S, Zong Q, Anderson-Luxford D, Song X, Lv Z, Lv C (2021). Mental health of college students during the COVID-19 epidemic in China. J Affect Disord.

[30] Li Y, Zhao J, Ma Z, McReynolds LS, Lin D, Chen Z, Wang T, Wang D, Zhang Y, Zhang J (2021). Mental health among college students during the COVID-19 pandemic in China: A 2-wave longitudinal survey. J Affect Disord.

[31] Zhuo L, Wu Q, Le H, Li H, Zheng L, Ma G, Tao H (2021). COVID-19-related intolerance of uncertainty and mental health among back-to-school students in Wuhan: the moderation effect of social support. Int J Environ Res Public Health.

[32] Wang C, Pan R, Wan X, Tan Y, Xu L, McIntyre RS, Choo FN, Tran B, Ho R, Sharma VK (2020). A longitudinal study on the mental health of general population during the COVID-19 epidemic in China. Brain Behavior And Immunity.

[33] Zhang WR, Wang K, Yin L, Zhao WF, Xue Q, Peng M, Min BQ, Tian Q, Leng HX, Du JL (2020). Mental health and psychosocial problems of medical health workers during the COVID-19 epidemic in China. Psychother Psychosom.

[34] Chang Jinghui YY, Wang Dong. Mental health status and its influencing factors among college students during the epidemic of COVID-19. Journal of Southern Medical University. 2020;40(2):(in Chinese). 10.12122/j.issn.1673-4254.2020.02.06. 10.12122/j.issn.1673-4254.2020.02.06 PMC7086131 32376528

[35] Yang Xiulan JW, Xia Guo, Wang Jun, Yu Wanwan. Analysis of the influence of social hierarchy background on depression and anxiety among college students. Chinese Journal Disease Control&Prevention. 2015;19(12):(in Chinese). 10.16462/j.cnki.zhjbkz.2015.12. 021.

[36] Sun Q-H, Su Y (2020). Psychological crisis intervention for college students during novel coronavirus infection epidemic. Psychiatry Research.

[37] Jiang Ruichen LA. Mental health status and its influencing factors of college students in Anhui Province during the coronavirus disease 2019 pandemic. J Environ Occup Med. 2020;37(9):(in Chinese). 10.13213/j.cnki.jeom.2020.20171

[38] Wu Yi HX, Qian Dongfu. Study on status of mental health among college students during COVID-19 epidemic. Chinese J Health Educ. 2020;36(8):(in Chinese). 10.16168/j.cnki.issn.1002-9982.2020.08.004.

[39] Cleofas JV, Rocha ICN (2021). Demographic, gadget and internet profiles as determinants of disease and consequence related COVID-19 anxiety among Filipino college students. Educ Inf Technol.

[40] Bao Y, Sun Y, Meng S, Shi J, Lu L (2020). 2019-nCoV epidemic: address mental health care to empower society. Lancet.

[41] Ayittey FK, Ayittey MK, Chiwero NB, Kamasah JS, Dzuvor C (2020). Economic impacts of Wuhan 2019-nCoV on China and the world. J Med Virol.

[42] Zhang Y-L, Liang W, Chen Z-M, Zhang H-M, Zhang J-H, Weng X-Q, Yang S-C, Zhang L, Shen L-J, Zhang Y-L (2013). Validity and reliability of patient health questionnaire-9 and patient health questionnaire-2 to screen for depression among college students in China. Asia-Pacific Psychiatry.

[43] Grant K, Marsh P, Syniar G, Williams M, Addlesperger E, Kinzler MH, Cowman S (2002). Gender differences in rates of depression among undergraduates: measurement matters. J Adolesc.

[44] Alansari BM (2006). Gender differences in depression among undergraduates from seventeen Islamic countries. Soc Behav Pers.

[45] Peng L, Zhang J, Li M, Li P, Zhang Y, Zuo X, Miao Y, Xu Y (2012). Negative life events and mental health of Chinese medical students: The effect of resilience, personality and social support. Psychiatry Research.

[46] Huang Yueqin DW, Liu Zhaorui et al. Psychosocial Aspects in Three Universities during SARS Epidemic in Beijing. Chinese Mental Health Journal. 2003;17(8):(in Chinese).

[47] Bruns DP, Kraguljac NV, Bruns TR (2020). COVID-19: Facts, Cultural Considerations, and Risk of Stigmatization. J Transcult Nurs.

[48] Xiang Y-T, Yang Y, Li W, Zhang L, Zhang Q, Cheung T, Ng CH (2020). Timely mental health care for the 2019 novel coronavirus outbreak is urgently needed. Lancet Psychiatry.

